# Improvement of the Dengue Virus (DENV) Nonhuman Primate Model via a Reverse Translational Approach Based on Dengue Vaccine Clinical Efficacy Data against DENV-2 and -4

**DOI:** 10.1128/JVI.00440-18

**Published:** 2018-05-29

**Authors:** Veronique Barban, Nathalie Mantel, Aymeric De Montfort, Anke Pagnon, Fabrine Pradezynski, Jean Lang, Florence Boudet

**Affiliations:** a Research and Development Department, Sanofi Pasteur, Marcy L'Etoile, France; Washington University School of Medicine

**Keywords:** flavivirus, dengue virus, nonhuman primate, protective immunity, animal models, preclinical study, vaccines, clinical trials, neutralizing antibodies

## Abstract

Recent data obtained with the live-attenuated tetravalent dengue CYD-TDV vaccine showed higher protective efficacy against dengue virus type 4 (DENV-4) than against DENV-2. In contrast, results from previous studies in nonhuman primates predicted comparable high levels of protection against each serotype. Maximum viral loads achieved in macaques by subcutaneous inoculation of DENV are generally much lower than those observed in naturally dengue virus-infected humans. This may contribute to an overestimation of vaccine efficacy. Using more-stringent DENV infection conditions consisting of the intravenous inoculation of 10^7^ 50% cell culture infectious doses (CCID_50_) in CYD-TDV-vaccinated macaques, complete protection (i.e., undetectable viral RNA) was achieved in all 6 monkeys challenged with DENV-4 and in 6/18 of those challenged with DENV-2, including transiently positive animals. All other infected macaques (12/18) developed sustained DENV-2 RNAemia (defined as detection of viral RNA in serum samples) although 1 to 3 log_10_ units below the levels achieved in control animals. Similar results were obtained with macaques immunized with either CYD-TDV or monovalent (MV) CYD-2. This suggests that partial protection against DENV-2 was mediated mainly by CYD-2 and not by the other CYDs. Postchallenge induction of strong anamnestic responses, suggesting efficient vaccine priming, likely contributed to the reduction of DENV-2 RNAemia. Finally, an inverse correlation between DENV RNA titers postchallenge and vaccine-induced homotypic neutralizing antibody titers prechallenge was found, emphasizing the key role of these antibodies in controlling DENV infection. Collectively, these data show better agreement with reported data on CYD-TDV clinical vaccine efficacy against DENV-2 and DENV-4. Despite inherent limitations of the nonhuman primate model, these results reinforce its value in assessing the efficacy of dengue vaccines.

**IMPORTANCE** The nonhuman primate (NHP) model is the most widely recognized tool for assessing the protective activity of dengue vaccine candidates, based on the prevention of postinfection DENV viremia. However, its use has been questioned after the recent CYD vaccine phase III trials, in which moderate protective efficacy against DENV-2 was reported, despite full protection against DENV-2 viremia previously being demonstrated in CYD-vaccinated monkeys. Using a reverse translational approach, we show here that the NHP model can be improved to achieve DENV-2 protection levels that show better agreement with clinical efficacy. With this new model, we demonstrate that the injection of the CYD-2 component of the vaccine, in either a monovalent or a tetravalent formulation, is able to reduce DENV-2 viremia in all immunized animals, and we provide clear statistical evidence that DENV-2-neutralizing antibodies are able to reduce viremia in a dose-dependent manner.

## INTRODUCTION

Dengue viruses (DENVs) are among the most important mosquito-borne pathogens that cause illness in humans. The incidence of disease caused by DENV has increased dramatically worldwide over recent decades (1). Four virus serotypes (DENV-1 to -4) circulate concomitantly in different regions of the world. These serotypes are responsible for infections that are either asymptomatic or able to cause a spectrum of clinical signs from classic dengue fever (DF) to dengue hemorrhagic fever (DHF) or dengue shock syndrome (DSS). These viruses have a sylvatic transmission cycle in nonhuman primates (NHPs), which may serve as a reservoir of epidemic DENV (2–4). Macaques (mainly Macaca mulatta and Macaca fascicularis) do not develop overt disease after experimental infection with human dengue virus strains. However, they are able to sustain viral replication in cell types relevant to human infection and develop a strong immune response (5–10). These monkey species therefore represent useful animal models for investigating various aspects of dengue virus infection and for evaluating candidate formulations of dengue vaccines (11–18). Among these formulations is the CYD-TDV dengue vaccine, a live-attenuated, tetravalent, chimeric yellow fever virus (YFV) developed by replacing the genes encoding the prM and E surface glycoproteins of the attenuated YFV 17D-204 strain by the respective counterparts from DENV-1, -2, -3, and -4 (14). The license for this vaccine (Dengvaxia) was based on one phase IIB (CYD23) and two large phase III (CYD14 and CYD15) efficacy trials in Asia-Pacific and Latin America in populations with a history of preexposure to flaviviruses (19–22). The pooled rate of overall efficacy for symptomatic dengue during the first 25 months of both phase III studies was 65.6% (95% confidence interval [CI], 60.7 to 69.9%) in subjects aged 9 to 16 years, irrespective of their dengue virus serostatus at baseline (20). A high level of protection was achieved against DENV-4 (83.2%; 95% CI, 76.2 to 88.2%), whereas the lowest level of efficacy was achieved against DENV-2 (47.1%; 95% CI, 31.3 to 59.2%). Complementary analyses have recently confirmed preliminary observations of a major role for prior dengue virus exposure in vaccine performance. Efficacy levels were statistically significant in seropositive participants but remained low to modest in those who were seronegative (S. Sridhar, A. Luedtke, E. Langevin, M. Zhu, M. Bonaparte, T. Machabert, S. Savarino, B. Zambrano, A. Moureau, A. Khromava, Z. Moodie, T. Westling, C. Mascarenas, C. Frago, M. Cortes, D. Chansinghakul, F. Noriega, A. Bouckenooghe, J. Chen, S. P. Ng, P. B. Gilbert, S. Gurunathan, and C. A. DiazGranados, submitted for publication).

During CYD-TDV development, the bioequivalence of vaccine batches across phase I, phase II, and phase III process steps was demonstrated by using the NHP model. The safety and immunogenicity profiles, consisting of transient, low-level vaccine viremia after the first inoculation and the induction of neutralizing antibodies (NAbs) against each of the four DENV serotypes after one or more vaccinations, were found to be consistent and in agreement with those observed in humans (14–16, 23–28).

Readouts of vaccine efficacy differ between humans and NHPs, i.e., protection against disease and viremia, respectively. Therefore, the capacity to protect against viremia after NHP challenge is considered more an indicator of vaccine potency (18). For this reason, and for cost and ethical considerations, the protective activity of CYD-TDV in macaques was evaluated only after the first stage of vaccine development using phase I vaccine lots. In this unique study, CYD-TDV was shown to confer almost full protection against wild-type (wt) DENV infection, with the percentage of aviremic animals ranging from 83% (DENV-1 and DENV-4) to 100% (DENV-2 and DENV-3) (15). Differences in rates of CYD-TDV efficacy between preclinical and clinical data indicated that protective activity in monkeys did not necessarily predict vaccine protection in humans. This offered a unique opportunity to improve the predictive value of the NHP model via a reverse translational approach.

A major limitation of the current NHP model is that viremia levels achieved after experimental dengue virus infection are generally lower than those observed following natural infection in humans (7, 29). Therefore, the milder challenge conditions may have resulted in an overestimation of the potency of CYD-TDV, especially against serotype 2. Most NHP challenge protocols for rhesus or cynomolgus macaques involve inoculation with 10^4^ to 10^6^ log_10_ PFU of DENV by the subcutaneous (s.c.) or, less frequently, the intradermal (i.d.) route. Each of these routes is considered to reflect virus delivery following the bite of an infected mosquito (30–33). Inoculation of DENV via the intravenous (i.v.) route may mimic direct injection into blood capillaries when a mosquito feeds on its host (34–37). Sustained levels of viremia accompanied by signs of dengue hemorrhages have been reported for rhesus macaques experimentally infected with DENV by this route (36).

The first objective of the work presented here was to develop a more virulent DENV-2 infection model in monkeys. The s.c., i.d., and i.v. inoculation routes were compared in cynomolgus macaques with two doses of DENV-2 (10^5^ and 10^7^ log_10_ 50% cell culture infectious doses [CCID_50_]). The most virulent conditions were then selected to reassess protection against viremia conferred by the CYD-TDV vaccine in cynomolgus macaques challenged with either DENV-2 or DENV-4. Animals were vaccinated with CYD-TDV clinical lots used in the efficacy trials, CYD14, CYD15 (phase III), and CYD23 (phase IIB), or with monovalent (MV) CYD-2 lots used for the preparation of CYD-TDV batches from CYD14 or CYD15 trials. Serological responses, including DENV-specific neutralizing antibodies (NAbs), proposed as potential correlates of protection in most flaviviral infections in both NHPs and humans (38–43) and antibody (Ab)-based enzyme-linked immunosorbent assay (ELISA) responses to YFV and DENV antigens were analyzed at different time points pre- and postchallenge. Using this more-virulent model, lower levels of protection against DENV-2 than against DENV-4 were observed, in better agreement with the clinical efficacy of the vaccines.

## RESULTS

### Selection of the most effective dengue virus infection conditions in NHPs.

**FIG 1:**
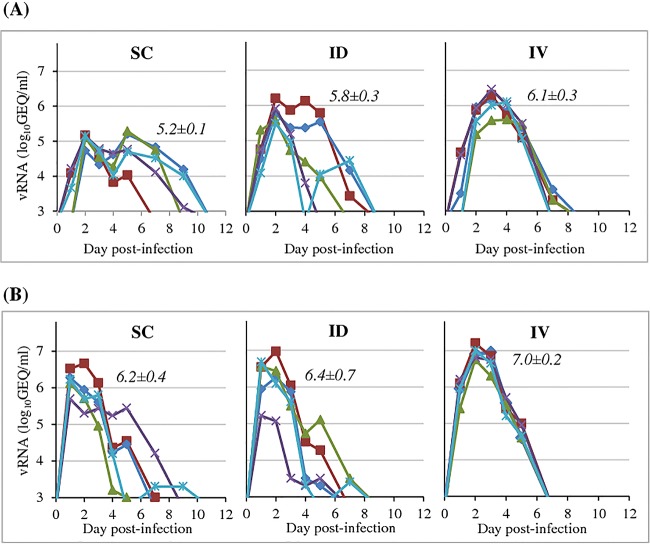
Selection of dose and route of DENV-2 inoculation (study A). NHPs were inoculated with 5.0 log_10_ CCID_50_ (A) or 7.0 log_10_ CCID_50_ (B) of DENV-2 strain 16681 via the route indicated in each graph: subcutaneous (SC), intradermal (ID), or intravenous (IV). The amount of viral RNA in plasma samples was quantified at different days postinoculation by a DENV-specific RT-qPCR assay. Each line represents the RNAemia curve for an individual macaque. Mean peak titers (log_10_ GEQ per milliliter) ± standard deviations are indicated at the top of each panel (for mean peak value comparisons, 7 log_10_ CCID_50_ > 5 log_10_ CCID_50_, *P* = 0.0001; i.v. > s.c., *P* = 0.048; i.v. > i.d., *P* < 0.0001; i.d. versus s.c., *P* = 0.11).

The aim of the first study was to establish the administration route/dose combination that produced the highest level of DENV-2 viremia in cynomolgus macaques. Six groups of macaques were inoculated with DENV-2 at 5.0 or 7.0 log_10_ CCID_50_ by either the s.c., i.d., or i.v. route (study A) (Table 1). Viral genomic RNA was quantified in plasma samples (RNAemia) from days 1 to 14 postinfection. Titers were compared to those obtained under standard experimental infection conditions (5.0 log_10_ CCID_50_ by the s.c. route) (Fig. 1). Individual RNAemia profiles elicited in animals infected by the i.d. or s.c. route were much more variable than those elicited in animals infected by the i.v. route, although the mean durations were similar (about 5 to 6 days). Furthermore, increasing the inoculum dose resulted in a shorter time to RNAemia irrespective of the administration mode. Both dose and route effects were demonstrated based on genomic RNA peak titers, with the highest titers being observed after i.v. high-dose inoculation (for mean peak value comparisons, 7 log_10_ CCID_50_ > 5 log_10_ CCID_50_, *P* = 0.0001; i.v. > s.c., *P* = 0.048; i.v. > i.d., *P* < 0.0001; i.d. versus s.c., *P* = 0.11). The magnitude of viremia was estimated by calculating the mean area under the curve (AUC) for RNAemia. Here too, the group inoculated i.v. with DENV-2 at a high dose displayed the highest mean AUC compared with the other tested conditions (mean AUCs ranging from 17.3 ± 0.9 to 14.0 ± 0.3 for i.v. administration of 7.0 log_10_ CCID_50_ and s.c. administration of 5.0 log_10_ CCID_50_, respectively; *P* < 0.05). This i.v./high-dose protocol thus appeared to fulfill our initial objective and was selected to reassess the immunoprotective efficacy of the CYD-TDV dengue vaccine against DENV-2 infection in further experiments.

**TABLE 1:** Design and sampling of DENV infection studies in cynomolgus macaques

Group (no. of animals)	Immunizations and challenges		Blood sampling days
	Virus (inoculation day[s])	Dose (CCID_50_); route of injection	
Study A			
A, B, C (5)	DENV-2 (0)	5 log_10_; s.c. (A), i.d. (B), i.v. (C)	1–14
D, E, F (5)	DENV-2 (0)	7 log_10_; s.c. (D), i.d. (E), i.v. (F)	1–14
Study B			
A, B, C (6)	CYD-TDV (0, 56)	5 log_10_/serotype/dose; s.c.	1–14, 28, 49, 84, 149, 305
	DENV-2 (315)	7 log_10_; i.v.	1–14, 28 postchallenge
D (6), E (7)	MV-CYD-2 (0, 56)	5 log_10_/serotype/dose; s.c.	1–14, 28, 49, 84, 149, 305
	DENV-2 (315)	7 log_10_; i.v.	1, 14, 28 postchallenge
F (7)	DENV-2 (315)	7 log_10_; i.v.	1–14, 28 postchallenge
Study C			
A, B (6)	CYD-TDV (0, 56)	5 log_10_/serotype/dose; s.c.	1–14, 28, 49, 84, 149, 305
	DENV-2 (315)	7 log_10_; i.v.	1–14, 28 postchallenge
C, D (6)	CYD-TDV (0, 56)	5 log_10_/serotype/dose; s.c.	1–14, 28, 49, 84, 149, 305
	DENV-4 (315)	7 log_10_; i.v.	1–14, 28 postchallenge

### Postvaccination CYD viremia and humoral responses.

In the second study, three groups of macaques were immunized by the s.c. route twice, at a 2-month interval (days 0 and 56), with human doses of CYD-TDV from clinical batch CYD14, CYD15, or CYD23 used in the corresponding phase III or phase IIB trials. This immunization schedule was used previously to document the preclinical bioequivalence of vaccine batches during CYD-TDV development and was shown to induce viremia and immune responses that were relatively close to those achieved in humans (28). Two additional groups were similarly immunized with the MV CYD-2 vaccine from CYD14 and CYD15 batches to evaluate the contributions of the other serotypes to virological, immunological, and protective responses elicited against serotype 2 in the tetravalent formulation (study B) (Table 1).

**FIG 2:**
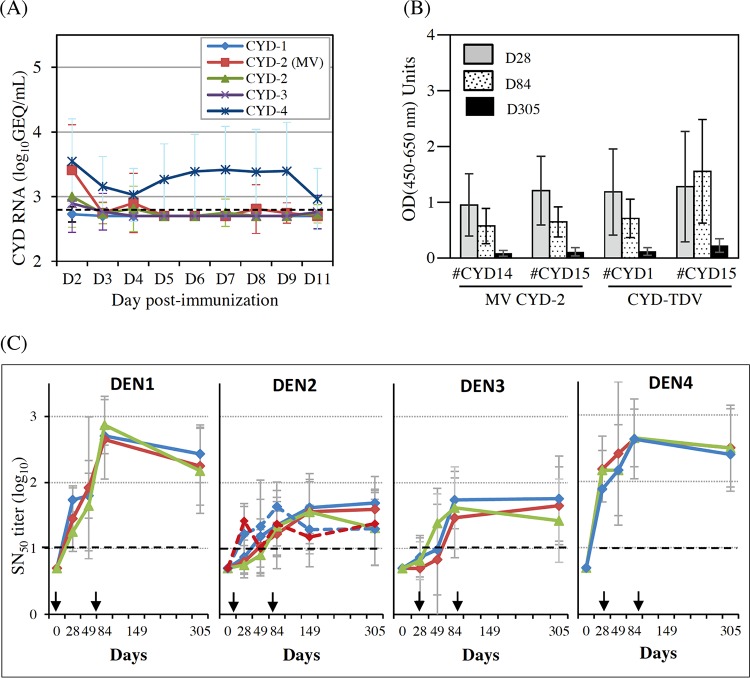
Viremia and immune responses induced after CYD immunization. (A) CYD RNAemia following s.c. immunization of NHPs with CYD-TDV or MV CYD-2 lots. Total RNA was extracted from plasma samples taken daily between days 2 and 11, and CYD titers were determined by using a one-step RT-quantitative PCR assay using serotype-specific primers (58). The mean titers ± standard deviations by serotype are indicated for each formulation (i.e., 3 groups with a mean of 18 monkeys for CYD-TDV and 2 groups with a mean of 13 monkeys for MV CYD-2). The dotted line indicates the lower limit of detection. (B) YFV NS1 ELISA antibodies were measured by a single-dilution ELISA using a purified His-tagged recombinant NS1 antigen (The Native Antigen Company Ltd.), and sera were tested at a 1:2,000 dilution. The positive cutoff was set at 0.01. GMTs and 95% CIs are shown. (C) Neutralizing antibodies to each of the four DENV serotypes (indicated at the top of each graph). Arrows indicate vaccination days. Plain curves indicate CYD-TDV groups. Dotted curves indicate MV CYD-2 groups. The dotted lines indicate the lower limit of quantification (LOQ = 10). GMTs and standard deviations are shown as logarithmic values. No significant difference was observed between groups. Blue, CYD14 lot; red, CYD15 lot; green, CYD23 lot.

The CYD viremia profiles assessed by reverse transcription-quantitative PCR (RT-qPCR) were consistent with those reported previously (44) (Fig. 2A; see also Table S1 in the supplemental material). Briefly, CYD-1, CYD-2, and CYD-3 genomic RNAs were transiently detected at low levels in the plasma of some CYD-TDV-vaccinated monkeys, soon after the first injection. In contrast, CYD-4 genomic RNA was consistently detected in all macaques. It was notable that the frequencies of CYD-2 genomic RNA detection were not statistically different between monovalent and tetravalent formulations (*n* = 9/13 and *n* = 7/12 serotype 2 RNA-positive monkeys for the MV CYD-2 and CYD-TDV formulations, respectively; *P* = 0.16) (Table S1).

As part of their replication cycle, CYD viruses secrete the hexameric form of YFV nonstructural protein 1 (NS1) common to the chimeric backbone of the four serotypes. YFV NS1-specific antibodies were monitored in a single-dilution ELISA as an indirect indicator of active CYD replication in MV CYD-2-vaccinated monkeys (Fig. 2B). YFV NS1 antibodies were detected at day 28 in all animals, including those negative for CYD-2 genomic RNA (*n* = 4/13). Antibody levels did not increase after the second vaccine dose (*P* = 0.18 for day 84 versus day 28) and were still detectable at day 305, i.e., 8 months after the second vaccine dose. No difference was observed between the two MV CYD-2 groups or between the MV CYD-2 and CYD TDV groups (*P* = 0.16). Using single-dilution commercial ELISA kits, a transient induction of DENV-specific IgM was detected in all groups. This peaked at day 28 and was followed by a gradual switch to IgG between the first and second doses. No difference was observed between the MV CYD-2 groups or between the CYD-TDV groups (data not shown).

Serum NAb titers to each DENV serotype were measured by a 50% seroneutralization (SN_50_) assay after dose 1 at days 28 and 49 and after dose 2 at days 84, 149, and 305 (Fig. 2C). Geometric mean titers (GMTs) increased after each immunization, whatever the serotype and formulation. The hierarchy of responses 1 month after the second dose (day 84) was similar to that observed previously in this model, namely, dominant responses against serotypes 1 and 4 and lower-level responses against serotypes 2 and 3 (28). No significant difference in terms of the numbers of responders or magnitudes of the response was detected between groups at later time points (*P* > 0.05 for all). SN_50_ responses to all serotypes persisted for about 8 months after the last immunization (day 305) in both MV CYD-2- and CYD-TDV-vaccinated groups, except for a slight decrease in DENV-1 SN_50_ titers between days 84 and 305 (−0.46 log_10_ units; *P* = 0.007). The response to serotype 2 plateaued at day 84 in MV CYD-2-vaccinated groups and slightly later in CYD-TDV-vaccinated groups, between days 84 and 149.

### Protection against DENV-2 challenge.

Eight months after the last vaccine dose (day 315), immunized monkeys were challenged, together with a group of nonvaccinated animals, under the optimized infection conditions (7.0 log_10_ CCID_50_ of the DENV-2 16681 strain by the i.v. route; study B) (Table 1). No overt clinical signs were recorded after challenge. An increase in glutamate-pyruvate transaminase (GPT) levels, mainly at day 7 postinfection, was observed in a small number of monkeys, distributed across the treatment groups. This effect, judged likely to be associated with DENV infection, was followed by a progressive decrease and a return to baseline values by day 28 postchallenge (see Table S2 in the supplemental material).

**FIG 3:**
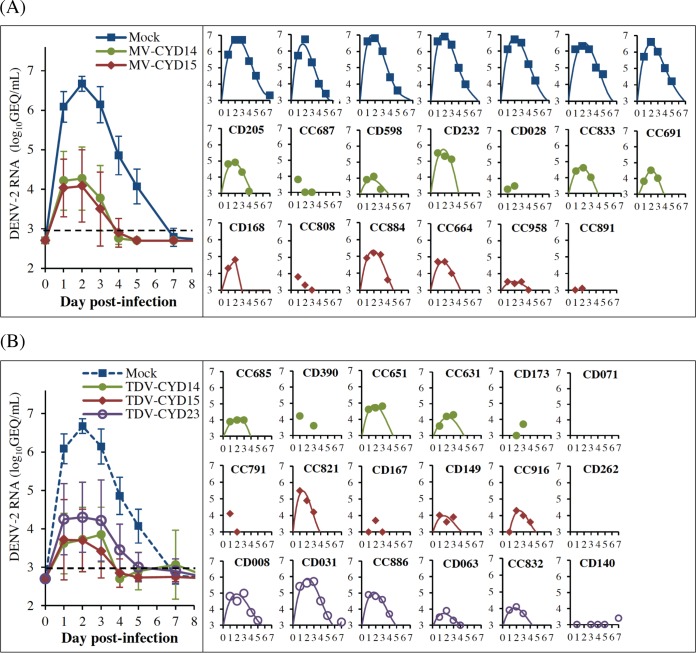
DENV-2 RNAemia after i.v. challenge with 7.0 log_10_ CCID_50_ of DENV-2 (study B). (A) Macaques vaccinated with MV CYD-2. (B) Macaques vaccinated with CYD-TDV. Total RNA was extracted from plasma samples at the indicated days postchallenge (the baseline sample collected at day −10 is referred to as day 0 for clarity), and DENV-2 genomic RNA was quantified by RT-qPCR. Left panels show mean RNA titers ± standard deviations for each formulation. Right panels show individual monkey RNA titers for each formulation (the animals are indicated at the top, except for mock-infected animals, which showed very similar profiles). Best mathematical curve fits are shown (*R*^2^ ≥ 0.95 for all). No line indicates that there was no or aborted viral replication (i.e., no increase in the genomic titer beyond day 1 and/or no development of full RNAemia curves, defined as at least 2 consecutive days above the quantification limit). The limit of quantification was 3.0 log_10_ GEQ/ml.

DENV-2 genomic RNA was quantified in plasma samples on a daily basis up to day 5 postchallenge. It was then measured every two (or three) days up to day 14, with a final time point at day 28 (Fig. 3A and B). Viral RNA was not detected in two animals from CYD-TDV groups (monkeys CD071 and CD262). Seven other monkeys (monkeys CC687, CC808, and CC891 in the MV CYD-2 groups and monkeys CD173, CC791, CD167, and CD140 in the CYD-TDV groups) showed evidence of abortive infection, meaning that there was no increase in genomic titers beyond day 1 and/or no development of full RNAemia curves (defined as at least 2 consecutive days with values above the quantification limit). DENV-2 propagation was observed in all other challenged animals vaccinated with either CYD-TDV (*n* = 12/18) or MV CYD-2 (*n* = 10/13) or nonvaccinated animals (*n* = 7/7), as shown by the early development of RNAemia curves. For both immunized and control animals, the time to peak RNAemia was 2 to 3 days on average. In comparison with nonvaccinated controls, infection was fully resolved (RNA titer lower than the limit of detection [LOD]) or nearly resolved (RNA titer ≤2× LOD) by day 5 postinfection in all vaccinated animals, except for one monkey in the CYD-TDV CYD23 group (monkey CD031) (*n* = 30/31). Low titers of DENV-2 RNA that did not exceed the LOD by 2-fold were also observed occasionally after day 5 and up to day 11 postchallenge in animals from the CYD-TDV CYD23 group and were likely to be not biologically significant (estimated infectious titers of 1 to 10 PFU/ml based on preestablished genome equivalent (GEQ)/PFU ratios [45]).

The ability of the CYD-2 vaccine, as a monovalent or tetravalent formulation, to control the magnitude of DENV-2 infection was analyzed after pooling individual virological parameters, i.e., RNAemia duration, peak titer, and AUC up to day 5, from either the three tetravalent or the two monovalent groups. The mean durations of DENV-2 RNAemia were similar between CYD-TDV and MV CYD-2 groups (3.0 ± 1.4 and 3.0 ± 0.8 days, respectively) but shorter than that for nonvaccinated animals (5.0 ± 0.0 days; *P* ≤ 0.001). In addition, there appeared to be no differences in mean AUCs and RNA peak titers in monkeys from the CYD-TDV and CYD-2 MV groups (AUCs, 3.9 ± 2.9 and 4.0 ± 2.4, respectively; peak titers, 4.2 ± 0.8 and 4.3 ± 0.7 log_10_ units; *P* = 0.62 and 0.55). However, these values were significantly lower than those for the nonvaccinated group (AUC, 13.6 ± 1.2; peak titer, 6.7 ± 0.2 log_10_ units; *P* ≤ 0.001 for all).

### Postchallenge ELISA responses to DENV antigens.

**FIG 4:**
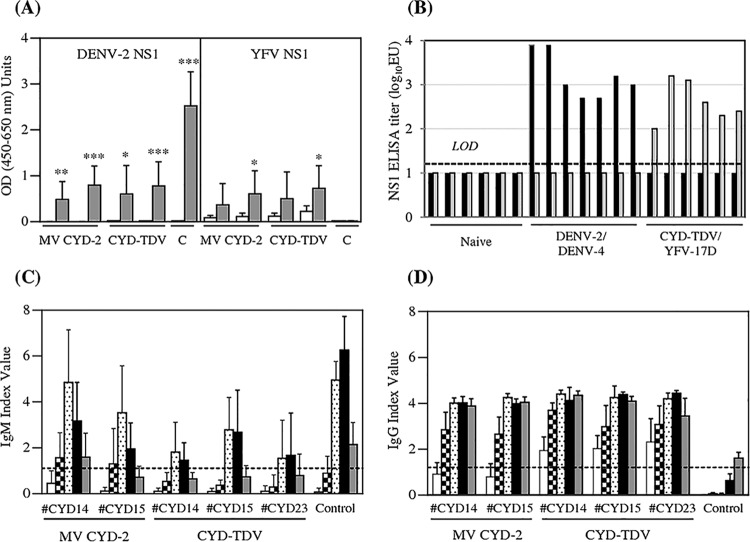
Evolution of vaccine-specific ELISA responses after DENV-2 challenge (study B). (A) YFV NS1- or DENV-2 NS1-specific IgG antibodies were detected by an ELISA using purified His-tagged recombinant NS1 antigens, as described in the legend of Fig. 2A. (B) Specificity of NS1 antibody capture ELISAs. Titers of antibodies in sera from NHPs immunized with DENV-2 (*n* = 6), DENV-4 (*n* = 1), YFV 17D (*n* = 1), or CYD-TDV (*n* = 5) or from naive animals (*n* = 6) were quantified over a wide range of 2-fold serial serum dilutions using DENV NS1 (black bars) or YFV NS1 (gray bars) soluble protein as the coating antigen. The titer of the reference serum was established in repeated, independent assays and calculated as the mean of the reciprocal serum dilution giving an OD_450–650_ of 1.0. The positive cutoff was set at 20 (1.3 log_10_ EU), and an arbitrary value of 10 (1 log_10_ EU) was assigned to all titers below this cutoff. (C and D) Levels of DENV-specific IgM (C) and DENV-specific IgG (D) were measured by an ELISA using commercial assays developed for diagnostics that have been proven to work with monkey sera. Coding of bars is as follows: white, day −10; squares, day 4; dots, day 7; black, day 14; grey, day 28. The dotted line indicates the positive threshold for human sera (index value of ≥1).

IgG serum antibody titers to secreted DENV-2 NS1 were monitored in the challenged monkeys by a single-dilution ELISA using His-tagged recombinant hexameric NS1 as the coating antigen (Fig. 4A). DENV-2 NS1 antibodies were detected in all monkeys, positive or not for DENV-2 genomic RNA, 1 month after challenge (day 28). Higher levels were reached in nonvaccinated than in vaccinated animals (*P* < 0.0001 for all), consistent with the reduction of DENV-2 RNA titers observed in all vaccinated groups. These levels were not different regardless of whether the CYD-2 vaccine was injected as a monovalent or a tetravalent formulation (*P* = 0.81), confirming that the immune response induced by heterologous serotypes in the tetravalent formulation had a low impact on protective activity against DENV-2. A slight increase in YFV NS1 titers after DENV-2 challenge was also observed for both monovalent and tetravalent CYD15 groups (*P* = 0.03 by a Wilcoxon paired *t* test). Although preliminary experiments conducted with few sera from naive animals inoculated with CYD-TDV or DENV-2 showed no cross-reactivity between DENV-2 and YFV soluble NS1 antigens (Fig. 4B), we cannot exclude a low level of cross-reactivity in these vaccinated animals submitted to high challenge doses.

DENV-specific IgM and IgG were measured before challenge (day −10) and at days 4, 7, 14, and 28 postchallenge by using human diagnostic ELISA kits (Focus Diagnostic) based on purified, inactivated dengue virus types 1 to 4 as capture antigens. These assays were thus also specific for CYD-TDV viruses that expose only the DENV envelope on their surface. In both control and vaccinated groups, DENV-specific IgM responses were increased at days 7 and 14 post-DENV-2 challenge compared to prechallenge responses (day −10) (Fig. 4C; see also Table S3A in the supplemental material). Of note, DENV-specific IgM was detected in all vaccinated animals on at least one time point, including monkeys with no detectable RNAemia (Table S3A). Compared with the control group, the IgM index values were significantly low for all vaccinated groups at day 14 (*P* < 0.01 for all, as determined by Dunnett adjustments by day) and in CYD-TDV CYD23 and CYD14 tetravalent groups at day 7 (*P* = 0.0041 and *P* = 0.0087, respectively), with lower-level DENV-2 viremia being observed for these animals.

DENV-specific IgG levels were also increased in all vaccinated animals compared to their prechallenge baseline levels (*P* < 0.01 for all, as determined by Dunnett adjustments by treatment for day −10/day 4) (Fig. 4D and Table S3B). This increase appeared earlier (day 4 instead of day 7) and was stronger in vaccinated groups than in nonvaccinated controls, at all time points.

### Neutralizing antibody responses after DENV-2 challenge.

**FIG 5:**
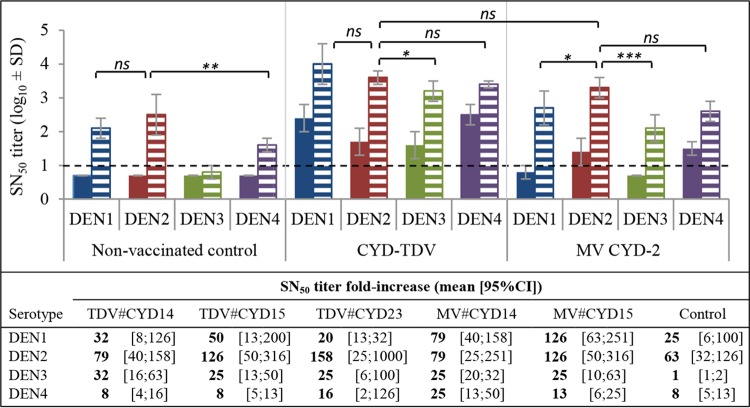
Neutralizing antibodies elicited after DENV-2 challenge (study B). NAb titers in serum samples collected at day −10 before DENV-2 challenge (plain bars) and at day 7 (CYD-TDV and control groups) or day 28 (MV CYD-2) after DENV-2 challenge (hatched bars) were quantified by an SN_50_ assay. As no significant difference in titers was observed between the three CYD-TDV or the MV CYD-2 groups (see Table S4 in the supplemental material), only one representative group is shown for each vaccine (CYD-TDV CYD14 and MV CYD-2 CYD14). A titer of half the limit of detection was assigned to negative samples, i.e., 0.7 log_10_ units. The fold increases in SN_50_ titers against each DENV serotype (days 1 to 4) are presented at the bottom for all groups. The dashed line indicates the limit of detection. Statistical comparisons were performed by using a mixed model with repeated measurements. *Post hoc* Tukey adjustments were performed by group for each comparison to ensure a final 5% risk. Additional comparisons are summarized in Table S4. ns, nonsignificant (*P* > 0.05); ***, *P* ≤ 0.001; **, *P* ≤ 0.01; *, *P* ≤ 0.05.

After DENV-2 challenge, nonvaccinated animals developed a strong homotypic neutralizing response to the infecting serotype, together with a strong heterotypic neutralizing response to DENV-1. In contrast, weak levels of heterotypic antibodies to DENV-4, and even lower levels of antibodies to DENV-3, were induced at the tested time point (Fig. 5).

In vaccinated groups, neutralizing antibody responses were homogeneous within groups treated with the same formulation (monovalent or tetravalent), and the same serotype hierarchy was observed (see Table S4 in the supplemental material). Statistical data described below relating to both MV CYD-2 and CYD-TDV CYD14 groups are representative of those for the other vaccine groups (Table S3C).

Just before challenge, monkeys vaccinated with MV CYD-2 lots still had detectable neutralizing antibodies to DENV-2 but also displayed heterotypic NAbs to serotype 4 (Fig. 5, right, plain bars). After DENV-2 challenge, the antibody profile induced in these animals showed similarity to that observed for nonvaccinated controls, except against serotype 3 (Fig. 5, right, hatched bars): NAb titers were strongly increased against both serotypes 2 and 1, while lower levels of heterotypic antibody responses were boosted against serotype 4 and against serotype 3.

A significant increase in the neutralizing antibody response to all four DENV serotypes was induced in the CYD-TDV-vaccinated monkeys further challenged with DENV-2 compared with their prechallenge baseline titers. NAb titers elicited against DENV-2 were not significantly different from heterotypic NAb titers induced against DENV serotypes 1, 3, and 4 (*P* > 0.5 for all) or from homotypic NAbs raised in MV CYD2-vaccinated challenged animals. The lowest booster effect on heterotypic responses was observed against serotype 4 (Fig. 5).

### Viremia and neutralizing antibody responses after DENV-4 challenge.

**FIG 6:**
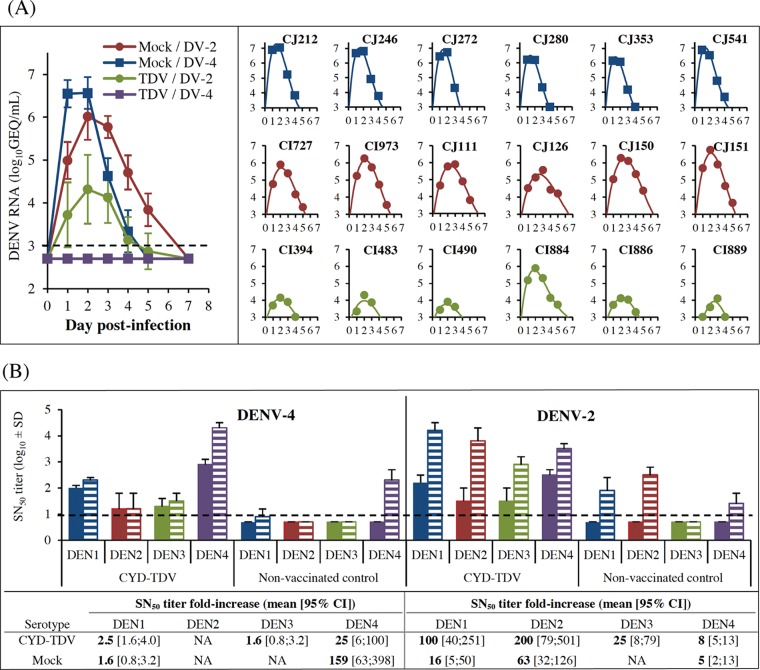
RNAemia and NAbs elicited after DENV-2 or DENV-4 challenge (study C). Twelve mock-inoculated and 12 CYD-TDV-vaccinated NHPs were separated into two groups and challenged by the i.v. route with 7.0 log_10_ CCID_50_ of either DENV-2 or DENV-4. (A) Viral RNA in plasma samples was quantified by RT-qPCR at the indicated days postchallenge. The left panel shows mean RNA titers ± standard deviations. The right panels show individual RNA titers, with the exception of DENV-4 mock-infected monkeys, as viremia was undetectable (animals are indicated at the top). Best mathematical curve fits are shown (*R*^2^ ≥ 0.95 for all). (B) NAb titers were quantified prechallenge (day −7) (plain bars) and postchallenge (day 14) (hatched bars) by an SN_50_ assay. Fold increases in SN_50_ titers against each DENV serotype (days 1 to 4) are presented at the bottom for all groups. The dashed lines indicate the detection threshold.

The aim of the third study was to assess the level of protection conferred by CYD-TDV against a DENV-4 challenge under the new conditions of i.v. high-dose infection (study C) (Table 1). Two groups of 6 macaques, either naive or vaccinated with CYD-TDV (lots were different from those tested in study B), were challenged with DENV-4 strain 1036 at 7.0 log_10_ CCID_50_ by the i.v. route (Fig. 6). Two similar groups challenged with DENV-2 were used as reference groups.

Control animals infected with DENV-4 showed homogeneous RNAemia profiles, with mean peak titers reaching 6.6 ± 0.4 log_10_ GEQ/ml, in a range similar to those observed for DENV-2-infected controls (Fig. 6A). However, RNA peak titers were achieved slightly faster and returned to baseline levels earlier after DENV-4 than after DENV-2 infection, indicating faster clearance. Overall, the duration of RNAemia was shorter with DENV-4 than with DENV-2.

DENV-4 genomic RNA was not detected in any of the vaccinated monkeys challenged with DENV-4, suggesting that infection with this serotype was fully prevented by CYD-TDV vaccination. A low-level antibody response against secreted DENV-4 NS1 was detected in 4 of 6 vaccinated monkeys by a quantitative ELISA, compared with nonvaccinated controls (GMTs, 1.6 ± 0.5 log_10_ ELISA units [EU] versus 4.0 ± 0.1 log_10_ EU, respectively). This might be due to the presence of residual NS1 in the DENV-4 inoculum or, alternatively, might be indicative of early, limited DENV-4 replication. However, since this response remained close to the positive cutoff of the assay (1.3 log_10_ EU) and no RNAemia was detected, CYD-TDV-vaccinated NHPs were regarded as being fully protected against DENV-4. As in study B, a significant, but not complete, reduction of DENV-2 RNAemia, was observed upon challenge with DENV-2 (*P* < 0.0001 for AUC and peak titer reductions). Also, all vaccinated animals developed lower DENV-2 NS1 antibody titers than those of nonvaccinated controls (mean titers of 3.4 ± 0.3 log_10_ EU versus 4.1 ± 0.1 log_10_ EU, respectively).

In the vaccinated NHPs, prechallenge NAb titers (day −7) against DENV-4 were about 20-fold higher (+1.3 log_10_ units) than those against DENV-2 (Fig. 6B). After infection with the homologous serotype, the increase in the homotypic response to DENV-2 was approximately 8 times higher than that of the response to DENV-4 (200-fold versus 25-fold, respectively). Heterotypic NAb responses to DENV-1, -2, or -3 were not significantly increased after DENV-4 infection of either vaccinated or nonvaccinated animals, as opposed to homotypic responses to DENV-4. As in study B, a strong increase in heterotypic responses to DENV-1, -3, and -4 together with homotypic responses to DENV-2 was observed after DENV-2 infection in both vaccinated and nonvaccinated animals.

### Immune parameters correlating with postchallenge DENV-2 viremia.

**FIG 7:**
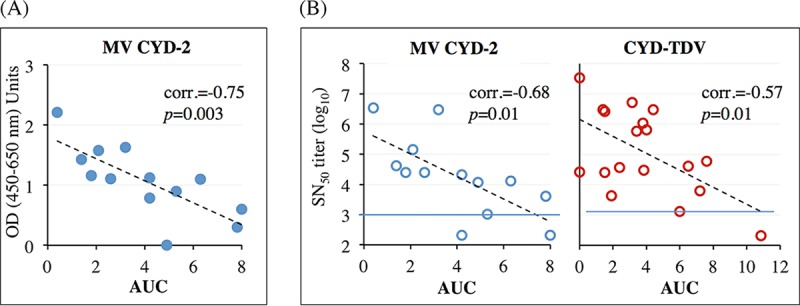
Parameters correlating with viremia after DENV-2 challenge (study B). Shown are data from assessments of statistical correlations between individual viremia AUCs of MV CYD-2 groups calculated within the first 5 days postchallenge and YFV NS1 ELISA titers at day 28 postvaccination (A) and prechallenge SN_50_ plateau titers to DENV-2 and viremia AUCs of either MV CYD-2 or CYD-TDV groups calculated within the 5 first days postchallenge (B). All investigated correlations are presented in Table S5 in the supplemental material. Red dotted lines indicate the assay threshold.

Correlation analyses were conducted on data from study B with the aim of identifying potential associations between prechallenge immune parameters and postchallenge DENV-2 viremia readouts (see comparisons in Table S5 in the supplemental material). The most statistically significant correlations are presented in Fig. 7.

Statistically significant inverse correlations were observed between the AUC (calculated within the first 5 days postchallenge) and (i) day 28 YFV NS1 antibodies for MV CYD-2 groups (Fig. 7A) and (ii) prechallenge DENV-2 SN_50_ titers at the plateau of the response (i.e., mean values of day 149 and day 305 titers) for MV CYD-2 (Fig. 7B) and CYD-TDV (Fig. 7C) groups.

## DISCUSSION

The aim of the present work was to reassess, in macaques, the protection conferred by the CYD-TDV vaccine, as a reverse translational post-phase III approach, under more stringent conditions of DENV infection than the ones employed previously. This strategy was based on the assumption that by using viremia levels that approximate those observed after natural dengue virus infection in humans, the new NHP model would have a better ability to predict CYD-TDV clinical efficacy against serotype 2.

The combined impact of three infection routes (s.c., i.d., and i.v.) and two DENV-2 doses (5 and 7 log_10_ CCID_50_) was first evaluated in cynomolgus macaques in order to select the most virulent challenge conditions. The average duration of viremia upon s.c. or i.d. inoculation at 5 log_10_ CCID_50_, and the shortened time to peak viremia at 7 log_10_ CCID_50_, was consistent with data from a previously reported meta-analysis of dengue virus infection in NHPs (46). The highest viremia levels were reached after i.v. inoculation of dengue virus type 2 at 7 log_10_ CCID_50_, with RNA titers being about 2 log_10_ units above those achieved after standard s.c. inoculation at 5 log_10_ CCID_50_. DENV-2 kinetics were shown to be highly homogeneous among animals and consistent between studies, in line with results described previously by Onlamoon et al. (36). However, in contrast to what was reported by Onlamoon et al., infection with DENV-2 (same strain, 16681) or DENV-4 did not result in cutaneous hemorrhages. Differences in animal genetics, sexes, and ages (i.e., young cynomolgus males in our study versus aged Indian rhesus females in the study by Onlamoon et al.) are likely to account for the discrepancies in results.

Full protection against viremia was achieved in all CYD-TDV-vaccinated macaques challenged with DENV-4 by the i.v. route, in line with the protective activity against this serotype described for NHPs under standard infection conditions, i.e., 5 log_10_ PFU via the s.c. route (15). In contrast, prevention of DENV-2 viremia was observed for only 20 to 30% of vaccinated monkeys, compared with 100% in previous NHP studies using the standard challenge protocol (15, 23). A different method was used in those early studies to assess vaccine protective activity: infectious virus titration by the focus-forming unit (FFU) method versus viral RNA quantification by an RT-qPCR assay in the present study. It is difficult to compare data from experiments conducted several years apart, but we have tried to estimate the number of DENV-2 aviremic animals postchallenge that would have been in our study if we had used an FFU assay. Knowing that the positive threshold was previously set at 1.7 log_10_ FFU/ml and using a preestablished GEQ/FFU ratio of 2.8 log_10_ units, prevention of DENV-2 viremia would have been detected in about 60 to 70% of vaccinated animals, suggesting that the low sensitivity of the FFU method compared with the PCR assay might also have contributed to overestimations of the protective activity of the CYD vaccine in previous NHP studies.

However, even if the FFU method had been applied to our study, CYD-TDV-induced protective activity against DENV-2 would have remained only partial, in better agreement with clinical efficacy data than with the previous NHP results (15, 23).

Despite the low number of animals that were fully protected against DENV-2 in the present study, our findings provide valuable information on the contribution of serotype 2 to vaccine efficacy. First, the similar rates of reduction of DENV-2 viremia achieved with monovalent and tetravalent formulations suggested that protective activity, even partial, was mediated mainly by CYD-2 *per se* and not by the other CYD serotypes. Second, the dominant neutralizing antibody responses raised by CYD-TDV against DENV-1 and DENV-4 that were still detectable at the time of challenge did not appear to affect vaccine protection against DENV-2.

All animals that were unprotected after DENV-2 challenge showed lower, but not delayed, RNA peak titers and a shorter duration of RNAemia than did the nonvaccinated control monkeys, suggesting that DENV-2 infection was rapidly counteracted by the vaccine-induced immune response. Early events in challenged vaccinated NHPs, such as a lower level and/or a more transient induction of DENV-specific IgM and DENV-2 NS1 antibodies as well as higher neutralizing antibody responses than in control animals, also support postinfection immune control. Moreover, we were able to establish a clear, statistically significant inverse correlation between neutralizing antibody titers before challenge and the DENV-2 viremia AUC. This observation confirmed the critical role of neutralizing antibodies in mediating protection against DENV. This was previously suggested for NHP, based on protection against challenge by passively transferred antibodies (38, 40), as well as for naturally infected humans (47, 48).

The strong increase in the titers of neutralizing antibodies against DENV-2 after a homologous viral challenge performed 10 months after the first immunization (and 8 months after the booster dose) revealed the capacity of the CYD vaccine to elicit immunological memory. The early induction of ELISA antibodies against YFV NS1 and the dengue virus-specific IgM-to-IgG switch postvaccination were the hallmarks of efficient immune priming that was probably a key driver in establishing immune memory. Interestingly, YFV NS1 antibody titers induced after the first vaccine dose were shown to be inversely correlated with the postchallenge DENV-2 viremia AUC in MV CYD-2 groups. Antibodies to the secreted form of YFV NS1 were assessed here as an indirect indicator of CYD replication. However, no correlation between CYD viremia postvaccination and DENV-2 viremia postchallenge in both MV CYD-2 groups could be demonstrated. This might reflect the higher sensitivity of the YFV NS1 Ab ELISA for detecting low, transient levels of CYD replication. However, given the multiple functions associated with flavivirus soluble NS1 (reviewed in reference 49), the meaning of this inverse correlation remains to be elucidated. Additional immune parameters that were not tested, such as the quality of neutralizing antibodies (avidity, IgG subclass, and epitope specificity, etc.) and the contribution of other types of functional antibodies (e.g., antibody-dependent cell-mediated cytotoxicity or phagocytosis) or of cell-mediated immunity, might also have been involved in mediating protection.

CYD-TDV vaccination did not confer sterilizing immunity in NHPs after DENV-2 or DENV-4 challenge, even for animals with undetectable or low-level/transient dengue virus RNAemia, which developed a strong anamnestic response, in agreement with previous observations of macaques immunized with CYD-TDV or other tetravalent dengue vaccine candidates (15, 50, 51).

Heterotypic antibody responses elicited in monkeys exposed to primary DENV infection are poorly documented in the literature (50, 52, 53). NHPs primarily infected with DENV-2 developed heterotypic neutralizing antibodies to DENV-1 and, to a lesser extent, to DENV-4. In contrast, animals vaccinated with MV CYD-2 developed heterotypic responses mainly to DENV-4. It is uncertain whether the distinct origins of the DENV-2 envelopes expressed by the challenge strain (strain 16681) and the MV CYD-2 vaccine (strain PUO-218) may account for the differences in heterotypic responses. In addition, CYD-2-primed monkeys that were further challenged with DENV-2 displayed broader and higher-level cross-reactive responses against the four serotypes. These responses were not different from those elicited in CYD-TDV-vaccinated monkeys further infected with DENV-2. Conversely, the neutralizing antibody response elicited upon DENV-4 infection was highly type specific in both naive and CYD-TDV-vaccinated NHPs. These findings were in agreement with the cross-reactive neutralizing antibody profile reported for CYD-TDV-vaccinated human subjects with no preexisting immunity to DENV (54). In this study, DENV-4 was shown to be neutralized mainly by type-specific antibodies, whereas DENV-2, together with DENV-1 and DENV-3, was neutralized mostly by cross-reactive antibodies. These data confirmed that some aspects of CYD-TDV heterotypic responses observed in dengue virus-naive humans could be reproduced in NHPs.

Although our improved NHP challenge model was able to replicate several immunological features observed for naturally dengue virus-infected humans showed a good correlation with data on the clinical efficacy of CYD-TDV against both dengue virus serotypes 2 and 4. However, it still displays some inherent limitations.

Lower efficacy and an increased risk of hospitalization have been reported for CYD-TDV-vaccinated subjects aged <9 years who were mainly seronegative for DENV (20, 59; Sridhar et al., submitted). Our study was conducted with flavivirus-negative monkeys, which should have theoretically been representative of this younger population. However, it was not possible to retrospectively predict such clinical data from the present preclinical results. This is because (i) the protective activity of CYD-TDV against DENV-1 and DENV-3 was not assessed in NHPs and (ii) the relative protective efficacy against DENV-2 and DENV-4 disease in subjects aged <9 years could not be statistically established due to the limited number of cases. Furthermore, the lack of clinical signs in our NHP model, which was not expected from data reported previously (36), remains a major limitation for predicting the potential risks of dengue disease after vaccination. This absence of disease despite a 2-log increase in viremia levels also strongly suggests that achieving high viral loads is not sufficient to induce the pathogenesis of human DENV strains in NHPs. Further exploration of genetic factors and immunological mechanisms underlying the resistance of NHPs to DENV-induced pathogenesis may help to refine this model and expand its translational relevance, as suggested by others (55, 56).

This is the first reverse translational approach to be used on a dengue vaccine and has enabled the current NHP model to be improved. Major assets include providing more conclusive data on the protective activity of CYD-TDV with a limited number of animals and identifying potential immune correlates of protection in monkeys. Additional studies will be needed once clinical efficacy data from other dengue vaccine candidates become available to confirm the predictive value of this monkey model.

## MATERIALS AND METHODS

### Study design.

The design of the three studies conducted with cynomolgus macaques, named studies A, B, and C, is described in Table 1. The immune protection studies B and C were conducted with groups of 6 or 7 monkeys. This number was inferred from a meta-analysis of data from 12 previous studies conducted at Sanofi Pasteur on CYD-TDV-immunized monkeys. According to this analysis, a sample size of 6 animals allows the detection of a difference of 0.6 log_10_ units in DENV-2-specific NAb titers between groups, with 80% statistical power and a 5% alpha risk.

### Animals, inoculations, and follow-up.

All studies were conducted with flavivirus-naive male Macaca fascicularis monkeys from Mauritius Island (Noveprim breeder), aged to 2 to 3 years and weighting 2.0 to 2.5 kg at the start of the study. Animals were randomized according to body weight and social affinities and were housed at Sanofi Pasteur (Marcy l'Etoile, France) in collective cages. Challenge studies were conducted at the Commissariat à l'Energie Atomique (CEA) (Fontenay-Aux-Roses, France) in biosecurity level A3 facilities. Animals were acclimatized in communicating individual cages for 1 month before DENV-2 or DENV-4 challenge. All animal protocols were approved by the Sanofi Pasteur internal animal care committee, and European guidelines for animal care were applied at both locations.

Challenge was done in a blind manner (no information on the administered vaccine was given to the CEA). s.c. and i.d. inoculations were done in the right scapula, and i.v. inoculations were done in the saphenous vein. Blood samples were taken from either unanesthetized or anesthetized macaques, and cumulated blood sample volumes per animal and per week did not exceed 10% of the total blood volume. Clinical examination of challenged animals was performed daily. Body weight and rectal temperature were recorded at each sampling time, and a complete blood count was performed: hematocrit, hemoglobin, red blood cells, thrombocytes, and white blood cells and their subsets (lymphocytes, monocytes, and granulocytes). Serum glutamate-pyruvate transaminase (GPT) and proteinemia levels were also determined.

### Products under test.

Immediately before inoculation into monkeys, clinical CYD-TDV vaccine doses (lyophilized purified product with a target formulation of 5/5/5/5, e.g., around 5.5 log_10_ CCID_50_ of each serotype) were resuspended in 0.5 ml of vaccine diluent, and the concentration of the MV CYD-2 lots (purified virus in liquid formulations) was adjusted to 5.5 log_10_ CCID_50_ per 0.5 ml of vaccine diluent. Two doses of each formulation were kept on ice during the inoculation time, and the titer was determined by a CCID_50_ assay at the end of the procedure. All titers were judged to conform.

The DENV-2 16681 and DENV-4 1036 wild-type strains were described previously (57). An internal Vero cell bank established from a cell clone derived from the ATCC CCL81 cell line was used for virus amplification. Frozen viral aliquots were thawed and diluted extemporaneously in phosphate-buffered saline (PBS). The inoculation volume by the s.c or i.d. route was 0.5 ml, and that by the i.v. route was 1 ml.

### Real-time PCR quantification.

Total RNA from plasma samples was extracted, and DENV RNA copy numbers were then determined by RT-qPCR, as described previously (58). The LOD and limit of quantification (LOQ) of these assays are 3.0 log_10_ GEQ/ml (i.e., 1 to 10 PFU/ml, depending on the serotype) and 3.3 log_10_ GEQ/ml, respectively. Samples for which no virus was detected were assigned a value of 2.7 log_10_ GEQ/ml (i.e., half the LOD) for GMT calculations.

### Neutralizing antibodies.

NAb responses to the DENV-1 16007, DENV-2 16681, DENV-3 16562, and DENV-4 1036 strains were monitored in 96-well tissue culture plates by an SN_50_ assay, as previously described (15) (16). The assay LOD, expressed as a reciprocal serum dilution, is 10, and a value of 5 was assigned to all samples below the LOD in calculations.

### ELISAs.

IgM and IgG responses to all four dengue virus serotypes were monitored by using the dengue virus IgM capture DxSelect and dengue virus IgG DxSelect human diagnostic kits (Focus Diagnostic), respectively, based on inactivated and purified dengue virus types 1 to 4 as capture antigens. The kits were used according to the manufacturer's instructions, and the positive threshold was set for an index value above 1, as for human samples. Negative index values were replaced by 0.001 in order to calculate geometric means and standard deviations. According to the manufacturer, the dengue virus IgG DxSelect ELISA kit shows 50% to 60% cross-reactivity with sera from YFV-vaccinated individuals. Since the CYD viruses do not express the YFV envelope genes (prM/E) and were purified, this potential cross-reactivity was not considered an issue for this study.

YFV and DENV-2 or DENV-4 NS1-specific IgG antibodies were measured by NS1 antibody capture ELISAs, using purified His-tagged recombinant hexameric NS1 protein (The Native Antigen Company Ltd.) as the coating antigen. Single-dilution assays (serum dilution of 1:2,000) were performed in study B, and results were expressed as the absorbance difference between 450 nm and 650 nm (OD_450–650_), corrected for blank readings. Positive OD cutoffs of 0.01 and 0.05, defined as the means plus 3 times the standard deviations calculated from a panel of naive monkey samples, were set for the YFV and DENV-2 NS1 proteins, respectively.

In study C, DENV-2 and DENV-4 NS1 antibody titers were established by regression analysis over a wide range of 2-fold serial serum dilutions, using reference NHP serum (serum from control animals infected with DENV-2 or DENV-4, respectively). The titer of the reference serum was established in repeated, independent assays and calculated as the mean of the reciprocal serum dilution giving an OD_450–650_ of 1.0. The positive cutoff was set at 20 (1.3 log_10_ EU), and an arbitrary value of 10 (1 log_10_ EU) was assigned to all titers below this cutoff. It should be noted that although the DENV-2 challenge inoculum may have contained a residual amount of secreted NS1, its impact on the total anti-NS1 antibody response postchallenge was minor.

### Statistical analyses.

Analyses of immune or virological parameters were carried out by using variance analysis models (parametric or nonparametric analyses depending on the tested readout). The percentages of positive monkeys (virus or neutralizing antibodies) were compared by using the Fisher test. When multiple comparisons were performed, *post hoc* adjustments were applied to have a global alpha risk of 5%. The association between postchallenge DENV-2 viremia and prechallenge vaccine-specific humoral responses was assessed by calculating Pearson correlation coefficients. All statistical analyses were performed with SAS v9.2 software. A margin of error of 5% was used for effects of the main factors.

## Supplementary Material

Supplemental material

## References

[1] Murray NE, Quam MB, Wilder-Smith A 2013 Epidemiology of dengue: past, present and future prospects. Clin Epidemiol 5:299–309. doi:10.2147/CLEP.S34440. 23990732 PMC3753061

[2] Inoue S, Morita K, Matias RR, Tuplano JV, Resuello RR, Candelario JR, Cruz DJ, Mapua CA, Hasebe F, Igarashi A, Natividad FF 2003 Distribution of three arbovirus antibodies among monkeys (Macaca fascicularis) in the Philippines. J Med Primatol 32:89–94. doi:10.1034/j.1600-0684.2003.00015.x. 12823631

[3] Kato F, Ishida Y, Kawagishi T, Kobayashi T, Hishiki T, Miura T, Igarashi T 2013 Natural infection of cynomolgus monkeys with dengue virus occurs in epidemic cycles in the Philippines. J Gen Virol 94:2202–2207. doi:10.1099/vir.0.055343-0. 23851439

[4] Vasilakis N, Cardosa J, Hanley KA, Holmes EC, Weaver SC 2011 Fever from the forest: prospects for the continued emergence of sylvatic dengue virus and its impact on public health. Nat Rev Microbiol 9:532–541. doi:10.1038/nrmicro2595. 21666708 PMC3321645

[5] Halstead SB, Shotwell H, Casals J 1973 Studies on the pathogenesis of dengue infection in monkeys. I. Clinical laboratory responses to primary infection. J Infect Dis 128:7–14. doi:10.1093/infdis/128.1.7. 4198027

[6] Halstead SB, O'Rourke EJ, Allison AC 1977 Dengue viruses and mononuclear phagocytes. II. Identity of blood and tissue leukocytes supporting in vitro infection. J Exp Med 146:218–229. doi:10.1084/jem.146.1.218. 195000 PMC2180735

[7] Marchette NJ, Halstead SB, Falkler WA Jr, Stenhouse A, Nash D 1973 Studies on the pathogenesis of dengue infection in monkeys. 3. Sequential distribution of virus in primary and heterologous infections. J Infect Dis 128:23–30. doi:10.1093/infdis/128.1.23. 4198025

[8] Marchette NJ, Halstead SB 1974 Immunopathogenesis of dengue infection in the rhesus monkey. Transplant Proc 6:197–201. 4208756

[9] Rosen L 1981 The use of Toxorhynchites mosquitoes to detect and propagate dengue and other arboviruses. Am J Trop Med Hyg 30:177–183. doi:10.4269/ajtmh.1981.30.177. 6111230

[10] Zompi S, Harris E 2012 Animal models of dengue virus infection. Viruses 4:62–82. doi:10.3390/v4010062. 22355452 PMC3280519

[11] Barban V, Munoz-Jordan JL, Santiago GA, Mantel N, Girerd Y, Gulia S, Claude JB, Lang J 2012 Broad neutralization of wild-type dengue virus isolates following immunization in monkeys with a tetravalent dengue vaccine based on chimeric yellow fever 17D/dengue viruses. Virology 429:91–98. doi:10.1016/j.virol.2012.03.007. 22542002

[12] Blaney JE Jr, Matro JM, Murphy BR, Whitehead SS 2005 Recombinant, live-attenuated tetravalent dengue virus vaccine formulations induce a balanced, broad, and protective neutralizing antibody response against each of the four serotypes in rhesus monkeys. J Virol 79:5516–5528. doi:10.1128/JVI.79.9.5516-5528.2005. 15827166 PMC1082773

[13] Eckels KH, Brandt WE, Harrison VR, McCown JM, Russell PK 1976 Isolation of a temperature-sensitive dengue-2 virus under conditions suitable for vaccine development. Infect Immun 14:1221–1227. 977127 10.1128/iai.14.5.1221-1227.1976 PMC415517

[14] Guirakhoo F, Pugachev K, Arroyo J, Miller C, Zhang ZX, Weltzin R, Georgakopoulos K, Catalan J, Ocran S, Draper K, Monath TP 2002 Viremia and immunogenicity in nonhuman primates of a tetravalent yellow fever-dengue chimeric vaccine: genetic reconstructions, dose adjustment, and antibody responses against wild-type dengue virus isolates. Virology 298:146–159. doi:10.1006/viro.2002.1462. 12093182

[15] Guirakhoo F, Pugachev K, Zhang Z, Myers G, Levenbook I, Draper K, Lang J, Ocran S, Mitchell F, Parsons M, Brown N, Brandler S, Fournier C, Barrere B, Rizvi F, Travassos A, Nichols R, Trent D, Monath T 2004 Safety and efficacy of chimeric yellow fever-dengue virus tetravalent vaccine formulations in nonhuman primates. J Virol 78:4761–4775. doi:10.1128/JVI.78.9.4761-4775.2004. 15078958 PMC387722

[16] Guy B, Barban V, Mantel N, Aguirre M, Gulia S, Pontvianne J, Jourdier TM, Ramirez L, Gregoire V, Charnay C, Burdin N, Dumas R, Lang J 2009 Evaluation of interferences between dengue vaccine serotypes in a monkey model. Am J Trop Med Hyg 80:302–311. 19190230

[17] Sariol CA, White LJ 2014 Utility, limitations, and future of non-human primates for dengue research and vaccine development. Front Immunol 5:452. doi:10.3389/fimmu.2014.00452. 25309540 PMC4174039

[18] WHO Expert Committee on Biological Standardization. 2013 Guidelines on the quality, safety and efficacy of dengue tetravalent vaccines (live, attenuated). World Health Organ Tech Rep Ser 979:1–366. 24340361

[19] Capeding MR, Tran NH, Hadinegoro SR, Ismail HI, Chotpitayasunondh T, Chua MN, Luong CQ, Rusmil K, Wirawan DN, Nallusamy R, Pitisuttithum P, Thisyakorn U, Yoon IK, van der Vliet D, Langevin E, Laot T, Hutagalung Y, Frago C, Boaz M, Wartel TA, Tornieporth NG, Saville M, Bouckenooghe A 2014 Clinical efficacy and safety of a novel tetravalent dengue vaccine in healthy children in Asia: a phase 3, randomised, observer-masked, placebo-controlled trial. Lancet 384:1358–1365. doi:10.1016/S0140-6736(14)61060-6. 25018116

[20] Hadinegoro SR, Arredondo-Garcia JL, Capeding MR, Deseda C, Chotpitayasunondh T, Dietze R, Muhammad Ismail HI, Reynales H, Limkittikul K, Rivera-Medina DM, Tran HN, Bouckenooghe A, Chansinghakul D, Cortes M, Fanouillere K, Forrat R, Frago C, Gailhardou S, Jackson N, Noriega F, Plennevaux E, Wartel TA, Zambrano B, Saville M 2015 Efficacy and long-term safety of a dengue vaccine in regions of endemic disease. N Engl J Med 373:1195–1206. doi:10.1056/NEJMoa1506223. 26214039

[21] Sabchareon A, Wallace D, Sirivichayakul C, Limkittikul K, Chanthavanich P, Suvannadabba S, Jiwariyavej V, Dulyachai W, Pengsaa K, Wartel TA, Moureau A, Saville M, Bouckenooghe A, Viviani S, Tornieporth NG, Lang J 2012 Protective efficacy of the recombinant, live-attenuated, CYD tetravalent dengue vaccine in Thai schoolchildren: a randomised, controlled phase 2b trial. Lancet 380:1559–1567. doi:10.1016/S0140-6736(12)61428-7. 22975340

[22] Villar L, Dayan GH, Arredondo-Garcia JL, Rivera DM, Cunha R, Deseda C, Reynales H, Costa MS, Morales-Ramirez JO, Carrasquilla G, Rey LC, Dietze R, Luz K, Rivas E, Miranda Montoya MC, Cortes Supelano M, Zambrano B, Langevin E, Boaz M, Tornieporth N, Saville M, Noriega F, CYD15 Study Group. 2015 Efficacy of a tetravalent dengue vaccine in children in Latin America. N Engl J Med 372:113–123. doi:10.1056/NEJMoa1411037. 25365753

[23] Guirakhoo F, Weltzin R, Chambers TJ, Zhang ZX, Soike K, Ratterree M, Arroyo J, Georgakopoulos K, Catalan J, Monath TP 2000 Recombinant chimeric yellow fever-dengue type 2 virus is immunogenic and protective in nonhuman primates. J Virol 74:5477–5485. doi:10.1128/JVI.74.12.5477-5485.2000. 10823852 PMC112032

[24] Guirakhoo F, Arroyo J, Pugachev KV, Miller C, Zhang ZX, Weltzin R, Georgakopoulos K, Catalan J, Ocran S, Soike K, Ratterree M, Monath TP 2001 Construction, safety, and immunogenicity in nonhuman primates of a chimeric yellow fever-dengue virus tetravalent vaccine. J Virol 75:7290–7304. doi:10.1128/JVI.75.16.7290-7304.2001. 11462001 PMC114964

[25] Guy B 2009 Immunogenicity of Sanofi Pasteur tetravalent dengue vaccine. J Clin Virol 46(Suppl 2):S16–S19. doi:10.1016/S1386-6532(09)70290-2. 19800561

[26] Guy B, Saville M, Lang J 2010 Development of Sanofi Pasteur tetravalent dengue vaccine. Hum Vaccin 6:696–705. doi:10.4161/hv.6.9.12739. 20861669

[27] Guy B, Almond J, Lang J 2011 Dengue vaccine prospects: a step forward. Lancet 377:381–382. doi:10.1016/S0140-6736(11)60128-1. 21277439

[28] Guy B, Barrere B, Malinowski C, Saville M, Teyssou R, Lang J 2011 From research to phase III: preclinical, industrial and clinical development of the Sanofi Pasteur tetravalent dengue vaccine. Vaccine 29:7229–7241. doi:10.1016/j.vaccine.2011.06.094. 21745521

[29] Vaughn DW, Green S, Kalayanarooj S, Innis BL, Nimmannitya S, Suntayakorn S, Endy TP, Raengsakulrach B, Rothman AL, Ennis FA, Nisalak A 2000 Dengue viremia titer, antibody response pattern, and virus serotype correlate with disease severity. J Infect Dis 181:2–9. doi:10.1086/315215. 10608744

[30] Bharati K, Rani R, Vrati S 2009 Evaluation of Japanese encephalitis virus DNA vaccine candidates in rhesus monkeys [Macaca mulatta]. Vaccine 27:10–16. doi:10.1016/j.vaccine.2008.10.050. 18996161

[31] Johnston LJ, Halliday GM, King NJ 2000 Langerhans cells migrate to local lymph nodes following cutaneous infection with an arbovirus. J Investig Dermatol 114:560–568. doi:10.1046/j.1523-1747.2000.00904.x. 10692118

[32] King NJ, Kesson AM 2003 Interaction of flaviviruses with cells of the vertebrate host and decoy of the immune response. Immunol Cell Biol 81:207–216. doi:10.1046/j.1440-1711.2003.01167.x. 12752685

[33] Verstrepen BE, Oostermeijer H, Fagrouch Z, van Heteren M, Niphuis H, Haaksma T, Kondova I, Bogers WM, de Filette M, Sanders N, Stertman L, Magnusson S, Lorincz O, Lisziewicz J, Barzon L, Palu G, Diamond MS, Chabierski S, Ulbert S, Verschoor EJ 2014 Vaccine-induced protection of rhesus macaques against plasma viremia after intradermal infection with a European lineage 1 strain of West Nile virus. PLoS One 9:e112568. doi:10.1371/journal.pone.0112568. 25392925 PMC4231036

[34] Griffiths RB, Gordon RM 1952 An apparatus which enables the process of feeding by mosquitoes to be observed in the tissues of a live rodent; together with an account of the ejection of saliva and its significance in malaria. Ann Trop Med Parasitol 46:311–319. doi:10.1080/00034983.1952.11685536. 13008362

[35] O'Rourke EJ, Fergus J 1956 Observations on pool and capillary feeding in Aedes aegypti (L.). Nature 177:1087–1088. doi:10.1038/1771087b0.

[36] Onlamoon N, Noisakran S, Hsiao HM, Duncan A, Villinger F, Ansari AA, Perng GC 2010 Dengue virus-induced hemorrhage in a nonhuman primate model. Blood 115:1823–1834. doi:10.1182/blood-2009-09-242990. 20042723 PMC2832810

[37] Styer LM, Kent KA, Albright RG, Bennett CJ, Kramer LD, Bernard KA 2007 Mosquitoes inoculate high doses of West Nile virus as they probe and feed on live hosts. PLoS Pathog 3:1262–1270. doi:10.1371/journal.ppat.0030132. 17941708 PMC1976553

[38] Hahn CS, French OG, Foley P, Martin EN, Taylor RP 2001 Bispecific monoclonal antibodies mediate binding of dengue virus to erythrocytes in a monkey model of passive viremia. J Immunol 166:1057–1065. doi:10.4049/jimmunol.166.2.1057. 11145685

[39] Hombach J, Solomon T, Kurane I, Jacobson J, Wood D 2005 Report on a WHO consultation on immunological endpoints for evaluation of new Japanese encephalitis vaccines, WHO, Geneva, 2-3 September, 2004. Vaccine 23:5205–5211. doi:10.1016/j.vaccine.2005.07.002. 16055233

[40] Lai CJ, Goncalvez AP, Men R, Wernly C, Donau O, Engle RE, Purcell RH 2007 Epitope determinants of a chimpanzee dengue virus type 4 (DENV-4)-neutralizing antibody and protection against DENV-4 challenge in mice and rhesus monkeys by passively transferred humanized antibody. J Virol 81:12766–12774. doi:10.1128/JVI.01420-07. 17881450 PMC2169078

[41] Markoff L 2000 Points to consider in the development of a surrogate for efficacy of novel Japanese encephalitis virus vaccines. Vaccine 18(Suppl 2):S26–S32. doi:10.1016/S0264-410X(99)00458-2. 10821970

[42] Mason RA, Tauraso NM, Ginn RK, O'Brien TC, Trimmer RW 1972 Yellow fever vaccine. V. Antibody response in monkeys inoculated with graded doses of the 17D vaccine. Appl Microbiol 23:908–913. 4624211 10.1128/am.23.5.908-913.1972 PMC380469

[43] Staples JE, Gershman M, Fischer M 2010 Yellow fever vaccine: recommendations of the Advisory Committee on Immunization Practices (ACIP). MMWR Recommend Rep 59:1–27. 20671663

[44] Guy B, Guirakhoo F, Barban V, Higgs S, Monath TP, Lang J 2010 Preclinical and clinical development of YFV 17D-based chimeric vaccines against dengue, West Nile and Japanese encephalitis viruses. Vaccine 28:632–649. doi:10.1016/j.vaccine.2009.09.098. 19808029

[45] Mantel N, Girerd Y, Geny C, Bernard I, Pontvianne J, Lang J, Barban V 2011 Genetic stability of a dengue vaccine based on chimeric yellow fever/dengue viruses. Vaccine 29:6629–6635. doi:10.1016/j.vaccine.2011.06.101. 21745519

[46] Althouse BM, Durbin AP, Hanley KA, Halstead SB, Weaver SC, Cummings DA 2014 Viral kinetics of primary dengue virus infection in non-human primates: a systematic review and individual pooled analysis. Virology 452–453:237–246. doi:10.1016/j.virol.2014.01.015. PMC4578724 24606701

[47] Buddhari D, Aldstadt J, Endy TP, Srikiatkhachorn A, Thaisomboonsuk B, Klungthong C, Nisalak A, Khuntirat B, Jarman RG, Fernandez S, Thomas SJ, Scott TW, Rothman AL, Yoon IK 2014 Dengue virus neutralizing antibody levels associated with protection from infection in Thai cluster studies. PLoS Negl Trop Dis 8:e3230. doi:10.1371/journal.pntd.0003230. 25329173 PMC4199527

[48] Katzelnick LC, Montoya M, Gresh L, Balmaseda A, Harris E 2016 Neutralizing antibody titers against dengue virus correlate with protection from symptomatic infection in a longitudinal cohort. Proc Natl Acad Sci U S A 113:728–733. doi:10.1073/pnas.1522136113. 26729879 PMC4725482

[49] Rastogi M, Sharma N, Singh SK 2016 Flavivirus NS1: a multifaceted enigmatic viral protein. Virol J 13:131. doi:10.1186/s12985-016-0590-7. 27473856 PMC4966872

[50] Koraka P, Suharti C, Setiati TE, Mairuhu AT, Van Gorp E, Hack CE, Juffrie M, Sutaryo J, Van Der Meer GM, Groen J, Osterhaus AD 2001 Kinetics of dengue virus-specific serum immunoglobulin classes and subclasses correlate with clinical outcome of infection. J Clin Microbiol 39:4332–4338. doi:10.1128/JCM.39.12.4332-4338.2001. 11724841 PMC88545

[51] Ambuel Y, Young G, Brewoo JN, Paykel J, Weisgrau KL, Rakasz EG, Haller AA, Royals M, Huang CY, Capuano S, Stinchcomb DT, Partidos CD, Osorio JE 2014 A rapid immunization strategy with a live-attenuated tetravalent dengue vaccine elicits protective neutralizing antibody responses in non-human primates. Front Immunol 5:263. doi:10.3389/fimmu.2014.00263. 24926294 PMC4046319

[52] Kochel TJ, Watts DM, Gozalo AS, Ewing DF, Porter KR, Russell KL 2005 Cross-serotype neutralization of dengue virus in Aotus nancymae monkeys. J Infect Dis 191:1000–1004. doi:10.1086/427511. 15717278

[53] Ito M, Katakai Y, Ono F, Akari H, Mukai RZ, Takasaki T, Kotaki A, Kurane I 2011 Serotype-specific and cross-reactive neutralizing antibody responses in cynomolgus monkeys after infection with multiple dengue virus serotypes. Arch Virol 156:1073–1077. doi:10.1007/s00705-011-0959-2. 21409446

[54] Henein S, Swanstrom J, Byers AM, Moser JM, Shaik SF, Bonaparte M, Jackson N, Guy B, Baric R, de Silva AM 2017 Dissecting antibodies induced by a chimeric yellow fever-dengue, live-attenuated, tetravalent dengue vaccine (CYD-TDV) in naive and dengue exposed individuals. J Infect Dis 215:351–358. doi:10.1093/infdis/jiw576. 27932620 PMC6392503

[55] Clark KB, Onlamoon N, Hsiao HM, Perng GC, Villinger F 2013 Can non-human primates serve as models for investigating dengue disease pathogenesis? Front Microbiol 4:305. doi:10.3389/fmicb.2013.00305. 24130557 PMC3795305

[56] Sariol CA, Pelegrino JL, Martinez A, Arteaga E, Kouri G, Guzman MG 1999 Detection and genetic relationship of dengue virus sequences in seventeen-year-old paraffin-embedded samples from Cuba. Am J Trop Med Hyg 61:994–1000. doi:10.4269/ajtmh.1999.61.994. 10674684

[57] Roehrig JT, Hombach J, Barrett AD 2008 Guidelines for plaque-reduction neutralization testing of human antibodies to dengue viruses. Viral Immunol 21:123–132. doi:10.1089/vim.2008.0007. 18476771

[58] Mantel N, Aguirre M, Gulia S, Girerd-Chambaz Y, Colombani S, Moste C, Barban V 2008 Standardized quantitative RT-PCR assays for quantitation of yellow fever and chimeric yellow fever-dengue vaccines. J Virol Methods 151:40–46. doi:10.1016/j.jviromet.2008.03.026. 18501437

[59] Arredondo-García JL, Hadinegoro SR, Reynales H, Chua MN, Rivera Medina DM, Chotpitayasunondh T, Tran NH, Deseda CC, Wirawan DN, Cortés Supelano M, Frago C, Langevin E, Coronel D, Laot T, Perroud AP, Sanchez L, Bonaparte M, Limkittikul K, Chansinghakul D, Gailhardou S, Noriega F, Wartel TA, Bouckenooghe A, Zambrano B for the CYD-TDV Dengue Vaccine Study Group 2 2 2018, posting date Four-year safety follow-up of the tetravalent dengue vaccine efficacy randomized controlled trials in Asia and Latin America. Clin Microbiol Infect, in press. doi:10.1016/j.cmi.2018.01.018. 29408333

