# Supplementary material for: Improvement of the Dengue Virus (DENV) Nonhuman Primate Model via a Reverse Translational Approach Based on Dengue Vaccine Clinical Efficacy Data against DENV-2 and -4
Source: J Virol. 2018 May 29;92(12):e00440-18. doi: 10.1128/JVI.00440-18 (PMC5974474; doi:10.1128/JVI.00440-18)
Supplement: Supplemental material [file supp_92_12_e00440-18__index.html]

Supplemental material 

# Improvement of the Dengue Virus (DENV) Nonhuman Primate Model via a Reverse Translational Approach Based on Dengue Vaccine Clinical Efficacy Data against DENV-2 and -4

## Supplemental material

- Supplemental file 1 -

  Table S1 (CYD RNAemia following immunization with CYD-TDV or MV CYD-2.)

  Table S2 (Serum glutamate-pyruvate transaminase levels pre- and post-DENV-2 challenge.)

  Table S3 (Statistical analyses supporting Fig. 4 and 5.)

  Table S4 (Neutralizing antibody titers of vaccinated monkeys before and after DENV-2 challenge.)

  Table S5 (Correlation analysis of postchallenge viremia with several prechallenge immune parameters.)

  PDF, 390K
