# Supplementary material for: Improvement of the Dengue Virus (DENV) Nonhuman Primate Model via a Reverse Translational Approach Based on Dengue Vaccine Clinical Efficacy Data against DENV-2 and -4
Source: J Virol. 2018 May 29;92(12):e00440-18. doi: 10.1128/JVI.00440-18 (PMC5974474; doi:10.1128/JVI.00440-18)
Supplement: Supplemental material [file JVI.00440-18_zjv012183587s1.pdf]

Supplementary Table S1. CYD RNAemia following immunization with CYD-TDV or MV CYD-2

CYD	Group	RNA-positive macaques	Genomic RNA peak	Detection duration
		N/total	$\log_{10}$ ±SD GEQ/mL / Peak Day	Days mean ± SD
CYD-1	CYD-TDV#CYD14	1 /6	2.8±0.2 / D2	0.2±0.4
	CYD-TDV#CYD15	0 /6	<2.8	NA
	CYD-TDV#CYD23	0 /6	<2.8	NA
CYD-2	MV #CYD15	5 /6	3.5±0.7 / D2	2.0±1.7
	MV #CYD14	4 /7	3.2±0.6 / D2	1.3±1.5
.	CYD-TDV#CYD14	5 /6	3.4±0.6 / D2	1.2±1.0
	CYD-TDV#CYD15	2 /6	2.8±0.3 / D2	0.3±0.5
	CYD-TDV#CYD23	2 /6	2.8±0.3 / D2	0.3±0.5
CYD-3	CYD-TDV#CYD14	2 /6	2.9±0.5 / D2	0.3±0.5
	CYD-TDV#CYD15	1 /6	2.9±0.4 / D2	0.2±0.4
	CYD-TDV#CYD23	2 /6	2.9±0.5 / D2	0.3±0.5
CYD-4	CYD-TDV#CYD14	6 /6	3.9±0.5 / D7-8	4.7±2.3
	CYD-TDV#CYD15	6 /6	3.7±0.5 / D5	5.7±1.4
	CYD-TDV#CYD23	6 /6	3.5±0.4 / D2	4.7±2.0

Supplementary Table S2. **Serum glutamate-pyruvate transaminase levels (UI/L) measured pre and post-DENV-2 challenge** Serum glutamate-pyruvate transaminase levels (UI/L) are indicated for monkeys from study B immunized with MV CYD-2 (top tables) or CYD-TDV (bottom table) vaccine formulations or for non-vaccinated monkeys (group F, bottom table). Bold characters: transaminase at least 2-fold above baseline levels. **(A)**, monovalent (MV) groups; **(B)** tetravalent (CYD-TDV) groups; **(C)**, control group

(A)

Group	Mk ID	D-10	D4	D7	D14	D28
**MV-CYD-2 #CYD15**	CD168	67	48	72	42	42
	CC808	93	47	46	35	31
	CC884	51	**184**	**265**	**209**	**107**
	CC664	76	65	57	45	39
	CC891	43	48	44	37	37
	CC958	44	**90**	**110**	**88**	58

Group	Mk ID	D-10	D4	D7	D14	D28
**MV-CYD-2 #CYD14**	CD205	37	**76**	**133**	**93**	73
	CC687	57	41	53	38	36
	CD598	43	39	41	34	47
	CD232	200	40	44	43	67
	CD028	44	65	66	61	54
	CC691	40	66	66	61	53
	CC833	74	48	44	42	43

(B)

Group	Mk ID	D-10	D4	D7	D14	D28
**CYD-TDV #CYD14**	CD390	55	81	90	72	28
	CC651	60	80	**181**	**152**	31
	CC631	35	**78**	**80**	65	**88**
	CD173	41	51	52	47	37
	CD071	42	60	77	60	36
	CC685	70	50	40	34	47
**CYD-TDV #CYD15**	CC791	78	48	53	56	46
	CD262	78	38	43	37	33
	CC821	42	64	78	55	48
	CD167	46	40	43	41	39
	CD149	68	62	89	58	45
	CC916	47	71	80	57	41
**CYD-TDV #CYD23**	CD008	36	50	46	47	40
	CD031	29	**64**	**76**	**65**	53
	CC886	64	42	47	45	37
	CD063	85	77	81	69	50
	CC832	38	48	56	39	30
	CD140	33	48	47	55	31

(C)

Group	Mk ID	D-10	D4	D7	D14	D28
**Control**	CF836	46	37	48	52	32
	CF839	57	54	64	50	41
	CF860	31	34	46	48	39
	CF868	54	55	59	57	43
	CF894	87	69	110	117	69
	CF950	85	84	89	67	64
	CF985	47	50	52	68	38

Supplementary Table S3. Statistical analyses supporting FIG.4 and FIG.5

(A) Post-challenge DENV-specific IgM **(Fig. 4B)**

Comparison	Estimate	Adjusted* p-value	Adjusted* lower limit 95% CI	Adjusted* upper limit 95% CI	Result
D-10 vs D4 for CYD23	-0.19	0.9512	-1.087	0.706	NS
D-10 vs D7 for CYD23	-1.445	0.1028	-3.106	0.216	NS
D-10 vs D14 for CYD23	-1.57	0.0374	-3.067	-0.074	S
D-10 vs D28 for CYD23	-0.69	0.0972	-1.474	0.094	NS
D-10 vs D4 for CYD_14_M	-1.117	0.0057	-1.946	-0.287	S
D-10 vs D7 for CYD_14_M	-4.394	<.0001	-5.932	-2.856	S
D-10 vs D14 for CYD_14_M	-2.711	<.0001	-4.097	-1.326	S
D-10 vs D28 for CYD_14_M	-1.142	0.0012	-1.868	-0.416	S
D-10 vs D4 for CYD_14_T	-0.435	0.5428	-1.332	0.461	NS
D-10 vs D7 for CYD_14_T	-1.715	0.0414	-3.376	-0.054	S
D-10 vs D14 for CYD_14_T	-1.357	0.0843	-2.854	0.139	NS
D-10 vs D28 for CYD_14_T	-0.546	0.24	-1.33	0.238	NS
D-10 vs D4 for CYD_15_M	-1.19	0.0064	-2.086	-0.293	S
D-10 vs D7 for CYD_15_M	-3.417	<.0001	-5.079	-1.756	S
D-10 vs D14 for CYD_15_M	-1.842	0.0121	-3.339	-0.346	S
D-10 vs D28 for CYD_15_M	-0.602	0.1726	-1.385	0.182	NS
D-10 vs D4 for CYD_15_T	-0.274	0.8475	-1.17	0.622	NS
D-10 vs D7 for CYD_15_T	-2.691	0.0008	-4.352	-1.03	S
D-10 vs D14 for CYD_15_T	-2.579	0.0004	-4.075	-1.082	S
D-10 vs D28 for CYD_15_T	-0.645	0.1316	-1.428	0.139	NS
D-10 vs D4 for control	-0.825	0.0519	-1.654	0.005	NS
D-10 vs D7 for control	-4.883	<.0001	-6.421	-3.346	S
D-10 vs D14 for control	-6.187	<.0001	-7.573	-4.802	S
D-10 vs D28 for control	-2.083	<.0001	-2.809	-1.357	S

* Dunnett Adjustement by treatment group

Comparison	Estimate	Adjusted* p-value	Adjusted* lower limit 95% CI	Adjusted* upper limit 95% CI	Result
CYD23 vs control for D-10	0.037	0.9994	-0.377	0.452	NS
CYD_14_M vs control for D-10	0.389	0.0576	-0.01	0.787	NS
CYD_14_T vs control for D-10	0.035	0.9996	-0.38	0.45	NS
CYD_15_M vs control for D-10	0.046	0.9984	-0.369	0.461	NS
CYD_15_T vs control for D-10	0.024	0.9999	-0.39	0.439	NS
CYD23 vs control for D4	-0.597	0.6192	-1.885	0.691	NS
CYD_14_M vs control for D4	0.681	0.4632	-0.557	1.918	NS
CYD_14_T vs control for D4	-0.354	0.9202	-1.642	0.934	NS
CYD_15_M vs control for D4	0.411	0.8655	-0.877	1.699	NS
CYD_15_T vs control for D4	-0.526	0.7201	-1.814	0.762	NS
CYD23 vs control for D7	-3.401	0.0041	-5.867	-0.936	S
CYD_14_M vs control for D7	-0.101	1	-2.47	2.268	NS
CYD_14_T vs control for D7	-3.134	0.0087	-5.599	-0.668	S
CYD_15_M vs control for D7	-1.42	0.4206	-3.885	1.046	NS
CYD_15_T vs control for D7	-2.168	0.1	-4.634	0.297	NS
CYD23 vs control for D14	-4.58	<.0001	-6.808	-2.351	S
CYD_14_M vs control for D14	-3.087	0.0026	-5.228	-0.946	S
CYD_14_T vs control for D14	-4.795	<.0001	-7.023	-2.567	S
CYD_15_M vs control for D14	-4.299	<.0001	-6.527	-2.071	S
CYD_15_T vs control for D14	-3.584	0.0008	-5.813	-1.356	S
CYD23 vs control for D28	-1.356	0.0148	-2.493	-0.218	S
CYD_14_M vs control for D28	-0.552	0.5424	-1.644	0.541	NS
CYD_14_T vs control for D28	-1.502	0.0062	-2.639	-0.365	S
CYD_15_M vs control for D28	-1.435	0.0093	-2.572	-0.298	S
CYD_15_T vs control for D28	-1.414	0.0105	-2.551	-0.277	S

* Dunnett Adjustement by Day

(B) Post-challenge DENV-specific IgG **(FIG 4.C)**

Comparison	Estimate	Adjusted* p-value	Adjusted* lower limit 95% CI	Adjusted* upper limit 95% CI	Result
D-10 vs D4 for CYD23	-0.767	0.0098	-1.375	-0.16	S
D-10 vs D7 for CYD23	-1.891	<.0001	-2.435	-1.346	S
D-10 vs D14 for CYD23	-2.132	<.0001	-2.72	-1.543	S
D-10 vs D28 for CYD23	-1.137	0.0016	-1.882	-0.392	S
D-10 vs D4 for CYD_14_M	-1.935	<.0001	-2.498	-1.373	S
D-10 vs D7 for CYD_14_M	-3.098	<.0001	-3.602	-2.593	S
D-10 vs D14 for CYD_14_M	-3.107	<.0001	-3.651	-2.562	S
D-10 vs D28 for CYD_14_M	-2.971	<.0001	-3.661	-2.281	S
D-10 vs D4 for CYD_14_T	-1.755	<.0001	-2.363	-1.148	S
D-10 vs D7 for CYD_14_T	-2.466	<.0001	-3.011	-1.922	S
D-10 vs D14 for CYD_14_T	-2.182	<.0001	-2.771	-1.594	S
D-10 vs D28 for CYD_14_T	-2.408	<.0001	-3.153	-1.662	S
D-10 vs D4 for CYD_15_M	-1.866	<.0001	-2.474	-1.259	S
D-10 vs D7 for CYD_15_M	-3.472	<.0001	-4.017	-2.927	S
D-10 vs D14 for CYD_15_M	-3.198	<.0001	-3.787	-2.61	S
D-10 vs D28 for CYD_15_M	-3.255	<.0001	-4.001	-2.51	S
D-10 vs D4 for CYD_15_T	-0.951	0.0012	-1.558	-0.343	S
D-10 vs D7 for CYD_15_T	-2.222	<.0001	-2.767	-1.677	S
D-10 vs D14 for CYD_15_T	-2.355	<.0001	-2.944	-1.767	S
D-10 vs D28 for CYD_15_T	-2.064	<.0001	-2.809	-1.318	S
D-10 vs D4 for control	-0.047	0.9985	-0.609	0.515	NS
D-10 vs D7 for control	-0.028	0.9997	-0.532	0.476	NS
D-10 vs D14 for control	-0.627	0.0202	-1.171	-0.082	S
D-10 vs D28 for control	-1.609	<.0001	-2.299	-0.919	S

* Dunnett Adjustement by treatment group

Comparison	Estimate	Adjusted * p-value	Adjusted* lower limit 95% CI	Adjusted* upper limit 95% CI	Result
CYD23 vs control for D-10	2.311	<.0001	1.416	3.205	S
CYD_14_M vs control for D-10	0.908	0.0355	0.048	1.767	S
CYD_14_T vs control for D-10	1.941	<.0001	1.047	2.836	S
CYD_15_M vs control for D-10	0.784	0.1018	-0.111	1.678	NS
CYD_15_T vs control for D-10	2.029	<.0001	1.134	2.923	S
CYD23 vs control for D14	3.816	<.0001	3.367	4.265	S
CYD_14_M vs control for D14	3.388	<.0001	2.957	3.819	S
CYD_14_T vs control for D14	3.497	<.0001	3.048	3.946	S
CYD_15_M vs control for D14	3.355	<.0001	2.907	3.804	S
CYD_15_T vs control for D14	3.757	<.0001	3.308	4.206	S
CYD23 vs control for D28	1.839	<.0001	1.275	2.402	S
CYD_14_M vs control for D28	2.27	<.0001	1.729	2.811	S
CYD_14_T vs control for D28	2.74	<.0001	2.177	3.304	S
CYD_15_M vs control for D28	2.43	<.0001	1.867	2.994	S
CYD_15_T vs control for D28	2.483	<.0001	1.92	3.047	S
CYD23 vs control for D4	3.031	<.0001	2.042	4.02	S
CYD_14_M vs control for D4	2.796	<.0001	1.846	3.746	S
CYD_14_T vs control for D4	3.65	<.0001	2.661	4.639	S
CYD_15_M vs control for D4	2.603	<.0001	1.614	3.593	S
CYD_15_T vs control for D4	2.932	<.0001	1.943	3.921	S
CYD23 vs control for D7	4.173	<.0001	3.797	4.55	S
CYD_14_M vs control for D7	3.977	<.0001	3.616	4.339	S
CYD_14_T vs control for D7	4.38	<.0001	4.003	4.756	S
CYD_15_M vs control for D7	4.228	<.0001	3.851	4.604	S
CYD_15_T vs control for D7	4.222	<.0001	3.846	4.599	S

* Dunnett Adjustement by Day

(C) Homotypic and heterotypic neutralizing antibody responses after DENV-2 challenge **(FIG. 5)**

Comparisons	Estimated difference (log10)	Adjusted p-value	Adjusted Lower 95%CI (log10)	Adjusted Upper 95%CI (log10)
DEN1 *vs.* DEN2	-0.37	0.06	-0.75	0.02
DEN1 vs. DEN3	1.34	<.0001	1.08	1.60
DEN1 vs. DEN4	0.56	0.02	0.11	1.00
DEN2 vs. DEN3	1.71	<.0001	1.45	1.97
DEN2 vs. DEN4	0.93	0.003	0.43	1.43
DEN3 vs. DEN4	-0.79	0.001	-1.14	-0.43

DEN1 *vs.* DEN2	0.42	0.27	-0.31	1.15
DEN1 vs. DEN3	0.83	0.01	0.23	1.44
DEN1 vs. DEN4	0.52	0.36	-0.52	1.56
DEN2 vs. DEN3	0.42	0.02	0.08	0.75
DEN2 vs. DEN4	0.10	0.79	-0.29	0.49
DEN3 vs. DEN4	-0.32	0.28	-0.88	0.25

DEN1 *vs.* DEN2	-0.59	0.02	-1.08	-0.10
DEN1 vs. DEN3	0.73	0.02	0.17	1.29
DEN1 vs. DEN4	-0.12	0.85	-0.65	0.42
DEN2 vs. DEN3	1.32	<.0001	1.12	1.53
DEN2 vs. DEN4	0.48	0.09	-0.08	1.03
DEN3 vs. DEN4	-0.85	0.02	-1.47	-0.22

Comparison DEN2	Estimated difference (log10)	p-value	Lower 95%CI (log10)	Upper 95%CI (log10)
TDV #CYD14 at D7 vs. MV #CYD14 at D28	0.12	0.30	-0.12	0.37
TDV #CYD14 at D7 vs. MV #CYD14 at D7	-0.20	0.22	-0.54	0.14
CYD-TDV at D7 vs. MV CYD-2 at D7	-0.10	0.43	-0.36	0.16

Supplementary Table S4. Neutralizing antibody titers of vaccinated monkeys before and after DENV-2 challenge. Titers were measured by SN50 assay at D-10 before challenge, and at D7 (CYD-TDV groups) or D28 (MV CYD-2 groups) after DENV-2 challenge. *SD: Standard Deviation*

Serotype		Pre-challenge titers		Post-challenge titers	
		$log_{10}SN_{50}$	SD	$log_{10}SN_{50}$	SD
CYD-TDV batches					
**DENV-1**	CYD-TDV #CYD14	**2.4**	**0.4**	**4.0**	**0.6**
	CYD-TDV #CYD15	**2.2**	**0.6**	**3.9**	**0.8**
	CYD-TDV #CYD23	**2.2**	**0.7**	**3.5**	**0.5**
**DENV-2**	CYD-TDV #CYD14	**1.7**	**0.4**	**3.6**	**0.2**
	CYD-TDV #CYD15	**1.6**	**0.3**	**3.7**	**0.4**
	CYD-TDV #CYD23	**1.3**	**0.6**	**3.6**	**0.5**
**DENV-3**	CYD-TDV #CYD14	**1.6**	**0.4**	**3.2**	**0.3**
	CYD-TDV #CYD15	**1.8**	**0.3**	**3.2**	**0.4**
	CYD-TDV #CYD23	**1.4**	**0.6**	**2.8**	**0.4**
**DENV-4**	CYD-TDV #CYD14	**2.5**	**0.3**	**3.4**	**0.1**
	CYD-TDV #CYD15	**2.5**	**0.2**	**3.4**	**0.2**
	CYD-TDV #CYD23	**2.4**	**0.9**	**3.2**	**0.4**
MV CYD-2 batches					
**DENV-1**	MV-CYD-2 #CYD14	**0.8**	**0.2**	**2.7**	**0.5**
	MV-CYD-2 #CYD15	**0.8**	**0.2**	**2.9**	**0.4**
**DENV-2**	MV-CYD-2 #CYD14	**1.4**	**0.4**	**3.3**	**0.3**
	MV-CYD-2 #CYD15	**1.3**	**0.5**	**3.4**	**0.2**
**DENV-3**	MV-CYD-2 #CYD14	**<1.0**	**NA**	**2.1**	**0.4**
	MV-CYD-2 #CYD15	**<1.0**	**NA**	**2.1**	**0.1**
**DENV-4**	MV-CYD-2 #CYD14	**1.5**	**0.2**	**2.6**	**0.3**
	MV-CYD-2 #CYD15	**1.5**	**0.1**	**2.9**	**0.3**

Supplementary Table S5. **Correlation Analysis of Post-Challenge Viremia with Several Pre-Challenge Immune Parameters.** DENV-2 viremia was estimated by calculation of the Area Under Curve (AUC) between day (D)1 and D5 after DENV-2 challenge, for each group. The average value per formulation (MV CYD-2, or CYD-TDV) was used in the comparisons. The following values were used for immune parameters: neutralizing antibodies (Nab), SN_{50} geometric mean titers; YF NS1 ELISA, mean Relative Units, Dengue IgG and IgM ELISA, mean Relative Units (as defined in the commercial assays), IgM to IgG ratio, mean ratio. Pearson's correlation coefficients are shown. *Bold characters: significant correlation coefficient (p-value ≤0.05)*

Parameter	Day	MV CYD-2 groups		CYD-TDV group	
		Correlation	*p*	Correlation	*p*
Nab (pre-challenge)	D149 and D305	**-0.68**	**0.01**	**-0.57**	**0.01**
YF NS1 ELISA	D28	**-0.75**	**0.003**	-0.33	0.29
	D84	-0.25	0.49	-0.17	0.62
	D305	-0.54	0.06	-0.52	0.08
IgG ELISA	D28	-0.26	0.38	-0.14	0.57
	D49	-0.35	0.24	-0.35	0.15
	D84	-0.32	0.28	-0.34	0.17
	D305	-0.46	0.11	-0.16	0.52
IgM ELISA	D28	-0.55	**0.05**	-0.03	0.92
	D49	-0.37	0.21	-0.26	0.30
	D84	-0.39	0.19	-0.30	0.22
	D305	0.31	0.30	-0.37	0.14
IgM/IgG	D28	-0.27	0.37	0.10	0.68
	D49	0.21	0.48	0.25	0.32
	D84	-0.29	0.34	-0.28	0.26
	D305	0.42	0.15	-0.32	0.19
